# Influences of Folate Supplementation on Homocysteine and Cognition in Patients with Folate Deficiency and Cognitive Impairment

**DOI:** 10.3390/nu12103138

**Published:** 2020-10-14

**Authors:** Yuka Hama, Tadanori Hamano, Norimichi Shirafuji, Kouji Hayashi, Asako Ueno, Soichi Enomoto, Miwako Nagata, Hirohiko Kimura, Akiko Matsunaga, Masamichi Ikawa, Osamu Yamamura, Tatsuhiko Ito, Yohei Kimura, Masaru Kuriyama, Yasunari Nakamoto

**Affiliations:** 1 Second Department of Internal Medicine, Faculty of Medical Sciences, University of Fukui, 23-3 Matsuokashimoaizuki, Eiheiji-cho, Yoshida-gun, Fukui 910-1193, Japan; kuronumayuka@gmail.com (Y.H.); sira@u-fukui.ac.jp (N.S.); fhsu-khayashi@kjb.biglobe.ne.jp (K.H.); maedaa@u-fukui.ac.jp (A.U.); weltraum@u-fukui.ac.jp (S.E.); gokuri@u-fukui.ac.jp (A.M.); iqw@u-fukui.ac.jp (M.I.); kapi@u-fukui.ac.jp (O.Y.); nakamoto-med2@med.u-fukui.ac.jp (Y.N.); 2 Department of Aging and Dementia (DAD), Faculty of Medical Sciences, University of Fukui, 23-3 Matsuokashimoaizuki, Eiheiji-cho, Yoshida-gun, Fukui 910-1193, Japan; 3 Life Science Innovation Center, Faculty of Medical Sciences, University of Fukui, 23-3, Matsuokashimoaizuki, Eiheiji-cho, Yoshida-gun, Fukui 910-1193, Japan; 4 Department of Neurology, Nakamura Hospital, 4-28 Tenno-cho, Echizen-city 915-0068, Japan; n.miwako@ca3.so-net.ne.jp; 5 Department of Radiology, Faculty of Medical Sciences, University of Fukui, 23-3, Matsuokashimoaizuki, Eiheiji-cho, Yoshida-gun, Fukui 910-1193, Japan; kimura@u-fukui.ac.jp; 6 Sukoyaka Silver Hospital, 93-6 Shimadera-cho, Fukui 910-3623, Japan; t-suko@fukui-sukoyaka-silver.or.jp; 7 Kimura Hospital, 57-25 Kitakanazu, Awara-city 919-0634, Japan; y-kimura@kimura-hospital.jp; 8 Ota Memorial Hospital,3-6-28, Okinogami-cho, Fukuyama 720-0825, Japan; kuriyama@shouwa.or.jp

**Keywords:** folate, homocysteine, vitamin B_12_, MMSE, hippocampal atrophy, MRI-VSARD

## Abstract

Although folate deficiency was reported to be associated with hyperhomocysteinemia, influence of folate supplementation on cognition remains controversial. Therefore, we explored the effects of folate supplementation on the cognition and Homocysteine (Hcy) level in relatively short periods in patients with folate deficiency and cognitive impairment. Enrolled 45 patients (mean age of 79.7 ± 7.9 years old) with folate deficiency (<3.6 ng/mL) with cognitive impairment underwent Mini-Mental State Examination (MMSE), and laboratory examinations, including folate, vitamin B_12_, and Hcy. The degree of hippocampal atrophy in MRI was estimated using a voxel-based specific regional analysis system for Alzheimer’s disease (VSRAD). Patients were administrated folate (5 mg/day), then Hcy, and MMSE score were re-examined after 28 to 63 days. Mean Hcy significantly decreased from 25.0 ± 18.0 to 11.0 ± 4.3 nmol/mL (*p* < 0.001). Average MMSE scores also significantly changed from 20.1 ± 4.7 to 22.2 ± 4.3 (*p* < 0.001). The degree of change in the MMSE score and basic Hcy or Hcy change was significantly positively correlated, while degree of hippocampal atrophy in MRI did not. Although several factors should be taken into account, folate supplementation ameliorated cognitive impairment, at least for a short period, in patients with folate deficiency.

## 1. Introduction

The increased prevalence of cognitive dysfunction is a major public health concern in aging populations worldwide [1]. The most common causes of cognitive impairment are Alzheimer’s disease (AD), vascular dementia (VaD), dementia with Lewy bodies (DLB) and frontotemporal dementia (FTD). Recently, the importance of vitamin deficiencies, including folate deficiency [2,3,4,5,6,7], for cognitive impairment has drawn attention.

**Figure 1:**
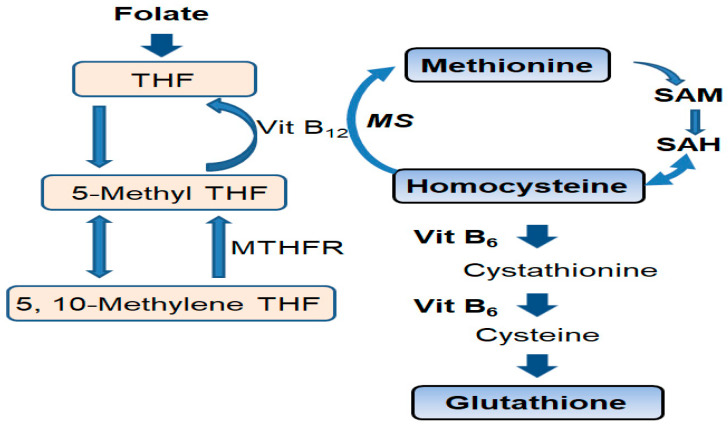
Homocysteine (Hcy) metabolic pathway. Folate and vitamin B_12_ are cofactors in the one carbon metabolism, during which they promote the remethylation of homocysteine. Vitamin B_12_ and folate deficiency inhibits the metabolism from Hcy to methionine, and causes hyperhomocysteinemia (HHCy). Vitamin B_6_ deficiency also inhibits the conversion of Hcy to cystathionine and causes hyperhomocysteinemia. SAM: S-adenosylmethionine; SAH: S-adenosylhomocysteine; Hcy: homocysteine; Vit B_6_: vitamin B_6_, Vit B_12_: vitamin B_12_; THF: tetrahydrofolate; 5-Methyl THF: 5-methyltetrahydrofolate; MTHFR: 5, 10-methylenetetrahydrofolate reductase; MS: methionine synthase.

As a homologue of cysteine, homocysteine (Hcy) is an intermediate in methionine metabolism (Figure 1) [8]. In one-carbon metabolism, folate is cofactor and vitamin B_12_ is a co-enzyme that promotes the remethylation of Hcy. Hcy is converted to cystathionine by vitamin B_6_. Thus, if folate is deficient, hyperhomocysteinemia (HHcy) can develop [7,8]. Vitamin B_12_ or B_6_ deficiencies can also cause HHcy (Figure 1). Hcy induces a high S-adenosylhomocysteine (SAH) level, which inhibits S-adenosylmethionine (SAM)-dependent methylation reactions (Figure 1) [9].

Recently, HHcy was suggested to lead to neurodegenerative disorders, including AD [6,8,10,11,12,13,14,15] and Parkinson’s disease (PD) [7,16]. HHcy is also associated with VaD [13,14,17]. Indeed, HHcy is associated with endothelial dysfunction [18,19], impaired nitric oxide activity [19], increased oxidative stress [11], cerebral microangiopathy [20], and gray matter atrophy in humans [21]. Disturbed cerebral blood flow due to atherosclerosis is an important factor for the disease progression of AD [22], and HHcy is a risk factor for stroke [14,23,24] and myocardial infarction [25].

Low concentrations of folate and HHcy were reported to be associated with cognitive impairment [2,3,4,5,10,12,13,14,26,27,28,29,30] and brain atrophy [6,21,30,31,32,33]. However, recovery of cognitive function by folate supplementation is controversial [5,6,27,34,35,36]. For example, some meta-analyses disclosed that folic acid and vitamin B_12_ supplementation reduced the risk of dementia [5,6]. However, other meta-analysis found that no conclusion can be drawn based on Hcy decline with the B vitamin supplementation including folate, due to the lack of studies focused on the patients with folate deficiency and cognitive impairment [36]. Durgo et al. reported the results of a three-year treatment with a folic acid (800 μg/day) to evaluate 3-year changes in cognitive function in subjects with relatively low folate levels (IQR 5.3 ng/mL), slightly elevated serum Hcy (IQR 13.0 nmol/mL), and normal cognitive function. The authors reported that the 3-year changes in memory, information processing speed, and sensorimotor speed were significantly better in the folic acid group than in the placebo group [37]. Hcy levels were also significantly reduced in the folic acid group. However, the effects of folate on patients with folate deficiency with cognitive impairment are not yet clear.

To clarify the issues described above, we investigated cognitive function, hippocampal atrophy and Hcy levels in patients with folate deficiency. The main purpose of our study was to explore the effects of folate supplementation on the Hcy level and cognition in relatively short periods in such patients.

## 2. Patients and Methods

### 2.1. Ethical Approval and Consent to Participate

This study was a prospective, open-label, multi-center, single-arm study. Participants who visited the dementia outpatient clinic at the University of Fukui Hospital or Nakamura Hospital between January 2008 and December 2018 were evaluated. All subjects and their caregivers present were interviewed by trained neurologists, or certified clinical psychologists. Age, sex, education (in years), medical history, lifestyle, and habits, drug or alcohol abuse, medication use, especially regular vitamin supplementation (B vitamins (B_1_, B_2_, B_6_, B_12_, and folic acid), vitamin C, vitamin E, and vitamin D), antihypertensive agents, anticholinergic drugs, and antipsychotics were examined. Neurological examination was performed by trained neurologists and the Mini-Mental State Examination (MMSE) [38] as a neuropsychological examination was carried out by certified clinical psychologists who were blinded to the purpose of the study. Laboratory tests for folate, vitamin B_1_, B_12_, Hcy, RBC, mean corpuscular volume (MCV), Hb, AST, ALT, Na, K, Cl, TSH, FT_3_, and FT_4_, and brain imaging by MRI were also performed. Among the participants, those with low folate concentrations (<3.6 ng/mL) were enrolled. Those with low concentrations of vitamin B_12_ (below 233 pg/mL) were excluded [39]. In this study, vitamin B_6_ was not measured. They were administered folate (5 mg/day: FOLIAMIN TABLETS^®^ containing 5 mg of folic acid, Nihon Pharmaceutical Co., Ltd., Tokyo, Japan) per os, and re-examined for the HDS-R, MMSE, and serum Hcy level 28 days to 63 days after starting folate supplementation. If the further MMSE follow-up was performed (6 months, 12 months and 24 months later), a generalized linear mixed model (GLMM) was applied to calculate the decline of MMSE score associated with folate. MCV was performed for 44. Ethics approval and consent to participate: The human clinical study protocol was approved by the ethics committee of the University of Fukui (20180092). Human rights: All human materials were obtained in accordance with the standards set forth in the Declaration of Helsinki principles of 1975, as revised in 2008 (http:// www.wma.net/en/10ethics/10helsinki/<http://www.wma.net/ en/10ethics/10helsinki/>).

### 2.2. Blood Sampling and Laboratory Tests

The folate and vitamin B_12_ levels were measured by the ADVIA Centaur XP Immunoassay System (Siemens Healthcare Diagnostics Manufacturing Limited, Dublin, Ireland) and its supporting kit (Siemens Healthcare Diagnostics Inc., Tarrytown, NY, USA) on the same day. Folate was quantitated by measuring the population of unoccupied folate-binding protein sites bound to the matrix using a conjugate of pteroic acid (a folate analogue) and alkaline phosphatase, as the signal-generating molecule, and substrate 4-methylumbelliferyl phosphate. A folate concentration <3.6 ng/mL in serum is considered to be folate deficiency. After folate supplementation, if the folate concentration was >20 ng/mL, value of folate was presented as <20. However, dilution procedure was performed in some patients (Patients 2, 3, 5, 6, 9, 11, 12, 13, 16, 19, 31, 33, 35, 37, 39, 40, and 44: Supplementary Table S1). The procedure to measure the folate level was identical in both groups, dilution was performed and dilution was not performed. The serum level of vitamin B_12_ was measured using the Siemens Healthcare Diagnostics Manufacturing Limited kit based on a microparticle enzyme immunoassay. The concentration of plasma Hcy was measured by atmosphere pressure ionization (API) 3200 LC-MS/MS system (SCIEX, Tokyo, Japan) using the LC-MS/MS system [40]. All laboratory tests were performed by clinical laboratory technicians who were blinded to the purpose of this study.

### 2.3. MRI Scans and Scoring of Brain Atrophy by VSRAD

All baseline MRI was performed on a 1.5- or 3.0-T GE Signa scanner at the University of Fukui Hospital or 1.5-T GE-Healthcare Optima MR 360 at Nakamura Hospital. By three-dimensional volumetric acquisition of a T1-weighted gradient echo sequence, a gapless series of thin sagittal sections was produced using the 3DFSPGR sequence (echo time/repetition time, 2.1/7.2 ms; flip angle, 25°; acquisition matrix, 256 × 256; 1 excitation: field of view, 24.0 cm, slice thickness, 1.4 mm) on the GE Signa scanner at the University of Fukui Hospital, and (echo time/repetition time, 4.4/12 ms; flip angle, 25°; acquisition matrix, 256 × 192; 1 excitation: field of view, 24.0 cm, slice thickness, 1.4 mm) on the 1.5-T GE-Healthcare Optima MR 360 at Nakamura Hospital.

Voxel-based morphometry (VBM) of MR images was performed as follows. Acquired MR images were analyzed by Statistical Parametric Mapping 2002 (SPM2) (Wellcome Department of Imaging Neuroscience, London, UK) which run on MATLAB (The MathWorks, Inc., Sherborn, MA, USA). To standardize the brain anatomically, individual brains were adjusted in three-dimensional space to a standard template brain, and the differences in brain size and shape were corrected to facilitate averaging among subjects. In the first anatomical standardization, 12-parameter affine transformation was applied. The MRI images after normalization were segmented into gray matter, white matter, cerebrospinal fluid, and other compartments using a maximum likelihood “mixed model” algorithm, a modified version of the clustering algorithm. The procedure of segmentation includes calculating, the Bayesian probabilities for each voxel belonging to each tissue class, based on a priori MRI information with heterogeneity adjustment. Gray matter images after segmentation were underwent affine and non-linear anatomical normalization with the priori-gray matter template. Gray matter images after anatomical standardization were smoothed with a 12-mm isotropic Gaussian kernel at full width at half maximum (FWHM), and a gray matter intensity spectrum was created using the partial volume effect. The gray matter intensity corresponds to a weighted average of gray matter voxels in a volume fixed by a smoothed kernel. Therefore, regional intensity can be considered almost equal to the concentration in gray matter. Each gray matter image was compared with the mean and S.D. of gray matter images of the 41 normal volunteers using voxel-by voxel z-score analysis following voxel normalization to global mean intensities; z-score = ([control mean] − [individual value])/(control S.D.). This software was designated as voxel-based specific region analysis for Alzheimer’s disease (VSRAD). Hippocampal atrophy on MRI was analyzed by this VSRAD z-score [41]. Z-score 0–1: no or minimal atrophy of the hippocampus, 1–2: mild atrophy of the hippocampus, 2–3: moderate atrophy of the hippocampus, 3–4: severe atrophy of the hippocampus. To estimate the degree of a region presenting marked atrophy in the entire brain (whole brain extent), the percentage of the coordinates with a z value transcending the threshold value of 2 in the whole brain was used. The MRI VSRAD z-score was calculated for 33 patients and whole brain extent was calculated for 26 patients. All MRI studies and VSRAD analysis by automatic computer software were performed by radiologists who were blinded to the purpose of this study.

### 2.4. Statistical Analysis

The main purpose of this study was to investigate the influence of folate supplementation on cognitive function in patients with low folate concentrations. The MMSE score following a normal distribution was analyzed by the Student’s *t*-test. Comparisons of Hcy levels after folate intake were also analyzed using the Wilcoxon signed-rank test as the data deviated from the normal distribution. Correlations between hippocampal atrophy on MRI and MMSE recovery by folate supplementation, and the correlation between the basic Hcy level or Hcy change and MMSE score recovery by folate administration were analyzed by Spearman’s rank correlation coefficient as the data deviated from the normal distribution. GLMM was applied to examine the influences of confounders, including age, sex, education and interval of follow up study. Missing values were treated by the list-wise deletion approach. Statistical analyses were performed using the SPSS statistics version 26 (IBM, Chicago, IL, USA). *p*-values < 0.05 were considered significant. The power of the data set was calculated using the free software G*Power 3.1 [42]).

## 3. Results

### 3.1. Folate Deficiency and Hyperhomocysteinemia

**Figure 2:**
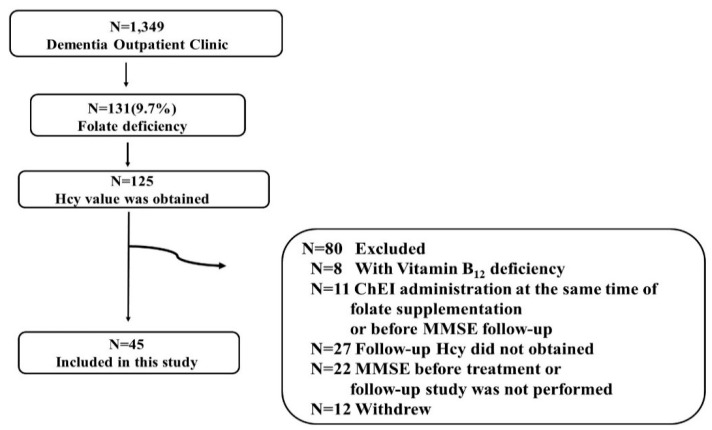
Flow chart detailing the derivation of the study sample. Of such patients, homocysteine (Hcy) was evaluated in 125 patients. Among them, 80 patients were excluded for the reasons described below: accompanying vitamin B_12_ deficiency (N = 8), choline esterase inhibitor (ChEI) administration at the same time as folate supplementation or before MMSE follow-up (N = 11), follow-up Hcy did not obtained (N = 27), MMSE before treatment or follow-up study was not performed (N = 22), or withdrew from the study (N = 12). In total, 45 patients were enrolled in this study.

During the evaluation period, 1349 patients visited our dementia outpatient clinic. Among them, 131 had folate deficiency (<3.6 ng/mL) (9.7%), 45 (28 men and 17 women) of whom were enrolled in this study. The process of selection of 45 patients among 131 patients with folate deficiency is shown in Figure 2. The included 45 patients and excluded 80 patients are listed in Supplementary Table S2. Among the 45 patients, five (patients 12, 18, 22, 25 and 38 in Supplementary Table S1) received methylcobalamin (1500 μg/day) administration for peripheral neuropathy before the entry into this study, and the dosage was not changed before and after folate therapy. No participants received other vitamin supplementation, including folate. The mean age was 79.7 ± 7.9 years old. The mean MMSE score before treatment was 20.1 ± 4.7. The mean concentration of folate was 2.7 ± 0.6 ng/mL (normal range 3.6–12.9), Hcy was 25.0 ± 18.0 nmol/mL (3.7–13.5), and vitamin B_12_ was 558.4 ± 406.5 pg/mL (233–914). The median education status (years) was 9 years (IQR 3) (Table 1). The detailed information of the participants is shown in Supplementary Table S1. A significant inverse correlation between the baseline folate concentration and Hcy level (0.006, R_s_ = −0.406) was observed (Supplementary Figure S1).

### 3.2. Brain Atrophy and Hyperhomocysteinemia, or Folate Deficiency

**Figure 3:**
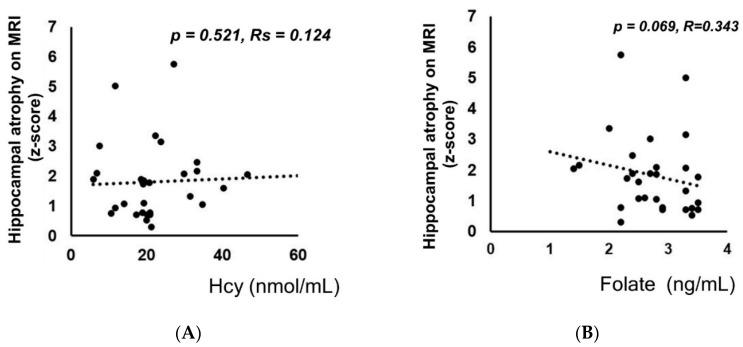
Plasma homocysteine (Hcy) levels or folate levels and the degree of hippocampal atrophy were not significantly correlated. There were no significant correlations between baseline Hcy levels and baseline hippocampal atrophy as estimated by the z-score produced by the voxel-based specific regional analysis system developed for the study of Alzheimer’s disease (VSRAD) (*p =* 0.521, Rs = 0.124). (**A**) The folate concentration and hippocampal atrophy based on the VSRAD z-score were not significantly correlated (*p =* 0.069, R = 0.343). (**B**) Spearman’s rank correlation coefficient was used for analysis because the data deviated from a normal distribution.

The mean z-score reflecting hippocampal atrophy on MRI in these patients was 1.91 ± 1.37 (Table 1). There were no significant correlations between baseline Hcy levels and hippocampal atrophy on MRI estimated by z-scores (*p =* 0.521, Rs = 0.124) (Figure 3A). Folate concentrations and hippocampal atrophy on MRI based on the VSRAD z-score were not significantly correlated (*p* = 0.069, Rs = 0.343) (Figure 3B). There were no significant correlations between baseline Hcy and extent of whole brain atrophy on MRI based on the entire brain (*p* = 0.971, Rs = −0.007) (Supplementary Figure S2A). Folate concentrations and extent of whole brain atrophy on MRI based on whole brain extent were not significantly correlated (*p =* 0.704, Rs = 0.077) (Supplementary Figure S2B).

### 3.3. Homocysteine Levels, and MMSE Scores Were Changed after Folate Supplementation

**Figure 4:**
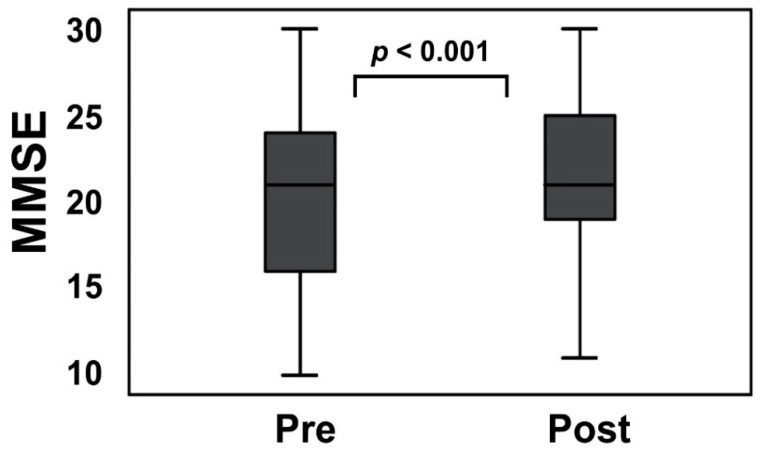
Folate supplementation significantly improved Mini-Mental State Examination (MMSE) in the short-term. The MMSE score improved from 20.1 ± 4.7 to 22.2 ± 4.3 (*p* < 0.001, effect size 0.59) 28 days to 63 days after folate supplementation. Bar: ± SD. The MMSE score following a normal distribution was analyzed by the Student’s *t*-test.

**Figure 5:**
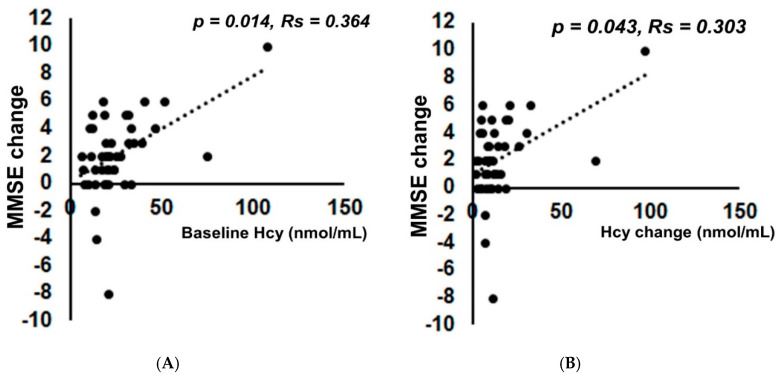
There was a significant positive correlation between MMSE score improvement after folate supplementation and the baseline homocysteine (Hcy) level or its reduction. (**A**) The degree of improvement of the MMSE score by folate supplementation and baseline homocysteine (Hcy) level were significantly correlated (*p* = 0.014, Rs = 0.364). (**B**) The degree of improvement of the MMSE score by folate supplementation and baseline Hcy reduction were significantly correlated (*p* = 0.043, Rs = 0.303). Spearman’s rank correlation coefficient was used for analysis because the data deviated from a normal distribution.

The mean Hcy level was markedly (*p* < 0.001) reduced from 25.0 ± 18.0 to 11.0 ± 4.3 nmol/mL (*p* < 0.001) by folate supplementation (Supplementary Figure S3). The mean folate level changed from 2.7 ± 0.6 ng/mL to 173.3 ± 257.2 ng/mL (*p* < 0.001). The mean MMSE scores after folate administration significantly changed from 20.1 ± 4.7 to 22.2 ± 4.3 (*p* < 0.001, effect size 0.59) (Figure 4). The power of the data set in MMSE after folate supplementation was estimated as 0.950. Confounders of age (*p* = −0.964, Rs = −0.003), sex (*p* = 0.224, Rs = 1.064), education (*p* = 0.719, Rs = −0.072) and interval of follow up study (*p* = 0.133, Rs = 0.058) had no effects on the MMSE change. There was a significant positive correlation between MMSE change and the baseline Hcy level (*p* = 0.014, Rs = 0.364) (Figure 5A).

**Figure 6:**
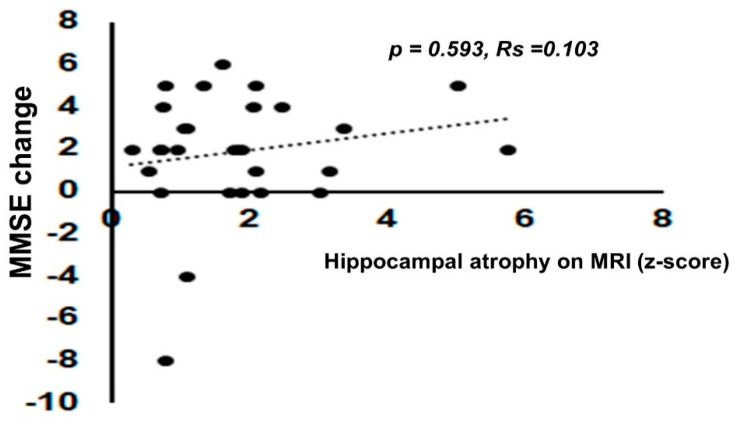
The degree of improvement in cognitive function by folate supplementation and hippocampal atrophy level were not significantly correlated. The degree of improvement of the MMSE score by folate supplementation and hippocampal atrophy as estimated by z-score by the voxel-based specific regional analysis system developed for the study of Alzheimer’s disease (VSRAD) (*p* = 0.593, Rs = 0.103). Spearman’s rank correlation coefficient was used for analysis because the data deviated from a normal distribution.

Of note, there was also a significant positive correlation between MMSE change and the Hcy change (*p* = 0.043, Rs = 0.303) (Figure 5B). There were no significant correlations between the degree of MMSE change by folate supplementation and the degree of hippocampal atrophy on MRI as estimated by the VSRAD z-score (*p* = 0.593, Rs = 0.103) (Figure 6). There was also no significant correlation between the degree of MMSE change by folate supplementation and degree of recovery of folate (Supplementary Figure S4). Long-term follow-up of the MMSE score at 6 months, 1 year, and 2 years later was performed by GLMM. Although the MMSE score decreased despite continued folate supplementation, it was gradual, as shown in Supplementary Figure S5.

### 3.4. The Folate Concentration and Mean Corpuscular Volume (MCV) Are Not Correlated

In this study, the average mean corpuscular volume (MCV) level was relatively high (94.6 ± 5.4 fL) (normal range 83.6 to 98.2 fL). However, no correlation between the folate concentration and MCV was observed (*p* = 0.595, R = 0.082) in this study (Supplementary Figure S6).

## 4. Discussion

In this study, low folate concentration was associated with HHcy. Folate intake also reduced plasma Hcy levels and the MMSE scores increased after folate supplementation regardless of the degree of hippocampal atrophy, at least for a short period, in patients exhibiting cognitive impairment with folate deficiency. Furthermore, the degree of MMSE score improvement was positively correlated with the baseline Hcy level and its reduction by folate supplementation. In previous studies, it remains unclear whether folate supplementation for individual patients is beneficial for cognition and the Hcy level. The folate concentration was reported to be low in general [12], whereas the Hcy level was high [12,14] in AD or mild cognitive impairment (MCI) patients. A low folate concentration was also found to be associated with increased Hcy and a decline in cognitive function [2,3,5,6,9,10,13,26,27,28,29,36,43,44]. Moreover, the folate concentration is inversely associated with the neocortex in the AD brain [30], whereas the Hcy level is positively associated with global brain atrophy [6], and cortical [21,32,33], subcortical [31,33] and hippocampal atrophy [33,35].

Possible mechanisms for the deleterious effects of Hcy on cognitive function are as follows: Hcy leads to DNA breakdown, oxidative damage, and the apoptotic process [7], in addition to as well as excitotoxic cell death by directly activating the neuronal NMDA receptor after formation of homocysteic acid [45]. Hcy is an indicator of methionine synthase (MS) activity and high Hcy levels can be an indicator of oxidative stress, which inhibits MS activity. MS activity is essential for D4 dopamine-mediated phospholipid methylation (PLM), which was proposed to play a central role in attention and cognition. Thus the improvement associated with folic acid supplementation and lower Hcy levels can be most directly explained by an improvement in MS activity and D4 dopamine receptor-mediated PLM [46]. Reactive oxygen radicals produced by Hcy is associated with amyloid β protein (Aβ pathology [45,47], thereby potentiating the neurotoxic effects of Aβ on its own or via homocysteic acid. Hcy may cause cerebral amyloid angiopathy and NO-mediated dysfunction of the endothelium in the cerebral vasculature and is associated with cardiovascular risk and its pathophysiology. It also activates tau kinases, including glycogen synthase kinase 3β (GSK3β) and cyclin dependent kinase 5 (Cdk5), to induce the cell cycle in neurons, causing neurofibrillary tangle deposition and/or cell death [8,15,45]. Hcy is converted to S-adenosylhomocysteine, which strongly inhibits transmethylation reactions. Many cellular methylation reactions may be inhibited, leading to the hyper-phosphorylation of tau [8]. Another report confirmed that folate deprivation significantly inhibits cell proliferation in the hippocampus, migration, survival, differentiation, transport of vesicles and plasticity of synapses [48].

Folate intake improved the mean MMSE scores, at least for a short period, in this study (Figure 5). The reason why cognitive function significantly improved for a short period after folate supplementation remains unclear. One possibility was mentioned above, folic acid supplementation and lower Hcy levels can improve MS activity and D4 dopamine receptor-mediated PLM, which were proposed to play a central role in attention and cognition [46]. Another possibility is that folate deficiency is one of the risk factors for depression [27]. Mood improvement may be beneficial for cognitive function. Other possibilities are as follows: practice effects or placebo effects. Another possibility is that because this study was performed at a dementia clinic, the doctor and health care providers provided other forms of care to stimulate brain function, including better daily living, such as a regular lifestyle, regular meals, reading newspapers and participating in social activities, which may have influenced the improvement in the MMSE score. However, as we had no controls, this cannot be confirmed.

Folate intake is associated with a lower long-term risk of dementia [49], the prevention of conversion of MCI to dementia [6], slower cognitive and clinical decline [50], and slower progression of brain atrophy [32,51], especially in the hippocampus, parahippocampal gyrus, interior parietal lobe, and retrosplenial cortex in MCI-patients [32]. Indeed, this study disclosed that the decline of MMSE score in the long term was generally slow (Supplementary Figure S5). Our basic study using a cell culture model of tauopathy demonstrated that Hcy-induced tau protein accumulation was reversed by the addition of folate to the media [8]. Li et al. previously reported that folate supplementation activated protein phosphatase 2A, an important tau phosphatase, and reduced phosphorylation levels of tau protein [52]. However, Doshi et al. confirmed that the direct pharmacological activity of folic acid improves endothelial function, instead of reducing Hcy [53]. Of note, the combined supplementation of folate, Vit B_6_ and B_12_ delayed gray matter atrophy, especially in patients with HHcy [32].

A meta-analysis by Ward et al. demonstrated that results, albeit not confirmatory of causation, provide an estimate of expected effects if the relationship was casual; an approximately 20% reduction in the risk of dementia by folic acid and vitamin B_12_ therapy [5]. Blasko et al. also reported that folate protects against the conversion of MCI to dementia [6]. Moreover, Tucker et al. found folate to be independently protective against a decline in the spatial copy score after adjusting for other vitamins or Hcy [43]. However, in previous randomized control trials, no differences in cognitive function estimated by MMSE scores between the vitamin supplementation group and control group were observed even though the Hcy level was reduced by folate [54,55]. In these studies, the mean folate levels were within the normal range. McCaddon and Miller stated after careful examination of meta-analyses that no conclusion can be made after the effects of Hcy reduction by B vitamins, including folate, on cognitive decline because the trials did not include individuals exhibiting such decline. Further definitive trials with older adults experiencing cognitive decline are needed [36].

Douaud et al. reported that higher Hcy levels were associated with faster grey matter atrophy, and that patients with HHcy treated using B vitamins (folate + B_6_ + B_12_) had a lower Hcy, which directly reduces grey matter atrophy, thereby slowing cognitive decline [32]. de Jager et al. also found that B vitamin treatment (folate + B_6_ +B_12_) slowed cognitive and clinical decline in MCI patients [49].

In this study, there was no significant correlation between the Hcy level and hippocampal atrophy estimated by the VSRAD z-score. Although the reason is not clear, one possible explanation is that there is a concomitant pathology with folate deficiency, including AD pathology, lewy body pathology or vascular pathology. However, although no significant correlation was found, the patients presenting lower folate levels presenting slightly higher VSRAD z-scores. The Hcy level and degree of recovery of the MMSE score were significantly positively correlated (Figure 5). This is partly consistent with the report by Douaud et al. that vitamin B_6_ + B_12_ + folic acid supplementation was effective in delaying gray matter atrophy in a group of patients with high Hcy levels. The degree of recovery of the MMSE score did not significantly correlate with the VSRAD z-score (Figure 6). Therefore, even with severe hippocampal atrophy, folic acid supplementation can be expected to restore cognitive function.

In this study, several patients exhibited high levels of folate after folate supplementation (Supplementary Table S1). It is generally accepted that folate supplementation is safe, but higher doses, such as the 5 mg can potentially cause adverse gastrointestinal effects, such as abdominal cramps, nausea and diarrhea, sleep disorders, irritability, rash, and lower seizure thresholds in patient taking anticonvulsants. Thus, monitoring folate levels in these patients and reducing the doses accordingly are necessary if the levels increase. Titrating down to a dosage that will maintain the folate levels and Hcy levels in the normal range is recommended.

In this study, only the folate level was measured, but it can increase to normal after a couple of folate-rich meals and/or taking folate vitamins. If the RBC folate level was measured, more patients with folate deficiency may have been found [37,56]. This should be performed in the future study. Moreover, this study was performed in Japan, which does not fortify its effect; therefore, the results in this study may not be applied to the countries with government-mandated folic acid fortification of flour [56].

In this study, MCV was generally high (94.9 ± 5.4 fL). This is consistent with previous reports of patients with folate or vitamin B_12_ deficiency [57,58]. The MCV value and folate level did not correlate with folate, consistent with the previous report [58].

There are several limitations in this study. First, the sample size was small, there was no blinded or control group, and the follow up period was short. Therefore, the effect size cannot be known exactly. Second, the depression scale (GDS15) was not examined. Vitamin B_6_, which also can be the cause of HHcy (Figure 1), was not measured. The MRI VSRAD z-score was obtained by 3 different scanners. Another limitation was that because this was performed at a dementia clinic, the doctor and health care providers provided other forms of care for the patients, including better daily living such as a regular lifestyle, regular meals, and reading the newspaper. Without doing a randomized study looking at other patients in the similar situation/category who did not have folate deficiency, then it cannot be automatically assumed that the folate is what improved the dementia scores and not some other forms of care given to the patients.

## 5. Conclusions

Folate supplementation may be useful to reduce Hcy levels and improve cognitive function in patients with folate deficiency in the short term regardless of hippocampal atrophy on MRI. The degree of MMSE score improvement correlated with the baseline Hcy levels and its reduction by folate supplementation. However, improvement of cognition needs to take into account several effects including exercise effects, placebo effects, or mood improvement with folic acid.

## Figures and Tables

**Table 1:** Demographics of 45 patients with folate deficiency.

Age (Mean ± SD)	79.7 ± 7.9
Male sex, n (%)	28 (62.2)
Education (Year) (Median (IQR))	9 (3)
MMSE (Mean ± SD)	20.1 ± 4.7
Folate (Mean ± SD), ng/mL	2.7 ± 0.6 (3.6–12.9)
Vitamin B12 (Mean ± SD), pg/mL	558.4 ± 406.5 (233–914)
Hcy (Mean ± SD), mmoL/mL	25.0 ± 18.0 (3.7–13.5)
MCV (Mean ± SD), fL	94.6 ± 5.4 (83.6–98.2)
MRI hippocampal atrophy z-score (Mean ± SD)	1.91 ± 1.37

Abbreviations: SD, standard deviation; IQR, interquartile range; MMSE, Mini-Mental State Examination; Hcy, homocysteine; (), normal range; MCV, mean corpuscular volume; MRI, Magnetic resonance imaging.

## Supplementary Materials

The following are available online at https://www.mdpi.com/2072-6643/12/10/3138/s1, Figure S1. Plasma folate concentrations and plasma homocysteine levels are inversely correlated. There was a significant inverse correlation between the baseline folate concentration and Hcy level (*p* = 0.006, Rs = −0.406). Spearman’s rank correlation coefficient was used because the data deviated from a normal distribution. Figure S2. Plasma homocysteine (Hcy) or folate and the degree of whole brain atrophy on MRI were not significantly correlated. There were no significant correlations between baseline Hcy and the extent of whole-brain atrophy on MRI based on the entire brain extent (*p* = 0.971, Rs = −0.007) (**A**) Folate concentrations and extent of whole-brain atrophy on MRI based on the entire brain were not significantly correlated (*p* = 0.704, Rs = 0.077) (**B**). Spearman’s rank correlation coefficient was used because the data deviated from a normal distribution. Figure S3. Folate supplementation improved plasma homocysteine (Hcy) levels. Folate supplementation significantly reduced Hcy levels from 25.0 ± 18.0 to 11.0 ± 4.3 nmol/mL (*p* < 0.001). Bar: ± SD. The Wilcoxon signed-rank test was used for the Hcy level because the data deviated from a normal distribution. Figure S4. MMSE change after folate supplementation and degree of recovery of folate were not correlated. There was no significant correlation between the degree of MMSE change after folate supplementation and degree of recovery of folate (*p* = 0.453, Rs = −0.118). Spearman’s rank correlation coefficient was used because the data deviated from a normal distribution. Figure S5. Long-term follow-up of Mini-Mental State Examination (MMSE) score. Long-term follow-up of the MMSE score at 6 months, 1 year and 2 years later by the generalized linear mixed model (GLMM). The decline in the MMSE score was gradual. M = months, Y = Years. Figure S6. There was no correlation between the folate concentration and mean corpuscular volume (MCV). The folate concentration and mean corpuscular volume (MCV) were not significantly correlated (*p* = 0.595, R = 0.082). Spearman’s rank correlation coefficient was used for analysis because the folate data deviated from a normal distribution. Table S1. Detailed information of 45 patients. Data for all 45 patients: age, sex, laboratory tests for folate, vitamin B12, and Hcy, and MMSE score. MCV was performed for 44, and MRI VSRAD was performed for 30. Abbreviations: Hcy, homocysteine; MMSE, Mini-Mental State Examination; VSRAD, voxel-based specific regional analysis system developed for the study of Alzheimer’s disease. Table S2. Comparison of Demographic data. Comparison of demographic data between patients included in this study (N = 45) and those not included (N = 80). Abbreviations: SD, standard deviation; MMSE, Mini-Mental State Examination; Hcy, homocysteine; (), normal range; VSRAD, voxel-based specific regional analysis system developed for the study of Alzheimer’s disease.
