# Supplementary material for: Influences of Folate Supplementation on Homocysteine and Cognition in Patients with Folate Deficiency and Cognitive Impairment
Source: Nutrients. 2020 Oct 14;12(10):3138. doi: 10.3390/nu12103138 (PMC7602498; doi:10.3390/nu12103138)
Supplement: Supplementary file 1 [file nutrients-12-03138-s001.pdf]

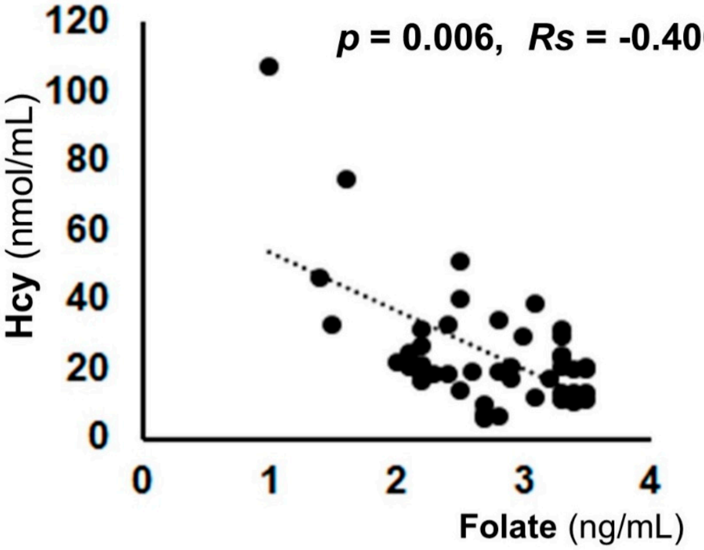

Supplementary Figure S1. Plasma folate concentrations and plasma homocysteine levels are inversely correlated. There was a significant inverse correlation between the baseline folate concentration and Hcy level ($p = 0.006$, Rs = -0.406). Spearman's rank correlation coefficient was used because the data deviated from a normal distribution.

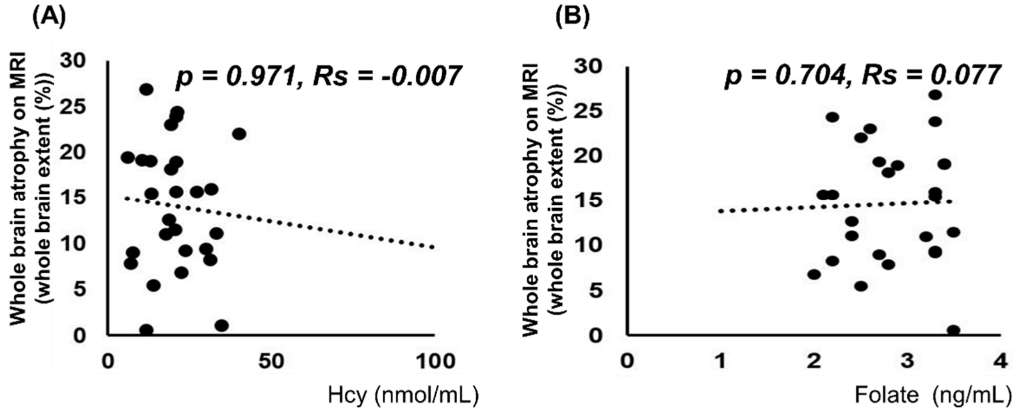

Supplementary Figure S2. Plasma homocysteine (Hcy) or folate and the degree of whole brain atrophy on MRI were not significantly correlated. There were no significant correlations between baseline Hcy and the extent of whole-brain atrophy on MRI based on the entire brain extent ($p = 0.971$, Rs=-0.007) (A). Folate concentrations and extent of whole-brain atrophy on MRI based on the entire brain were not significantly correlated ($p = 0.704$, Rs=0.077). Spearman's rank correlation coefficient was used because the data deviated from a normal distribution.

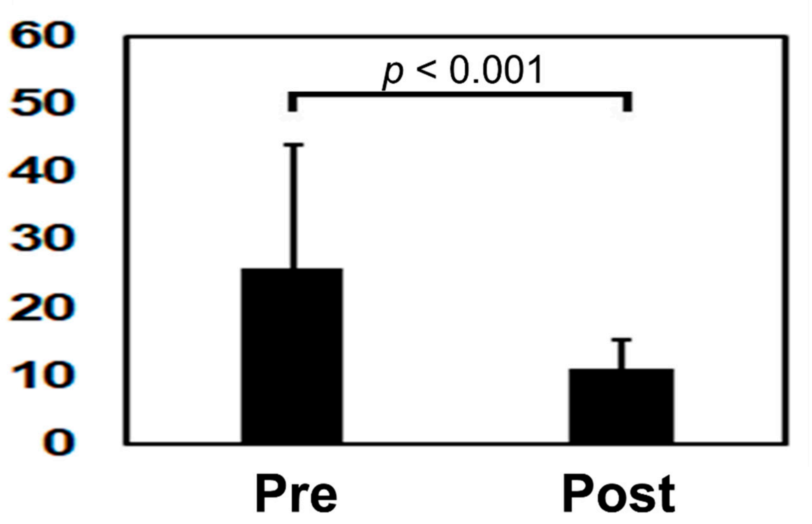

Supplementary Figure S3. Folate supplementation improved plasma homocysteine (Hcy) levels. Folate supplementation significantly reduced Hcy levels from 25.0 ± 18.0 to 11.0 ± 4.3 nmol/mL ($p < 0.001$). Bar: ± SD. The Wilcoxon signed-rank test was used for the Hcy level because the data deviated from a normal distribution.

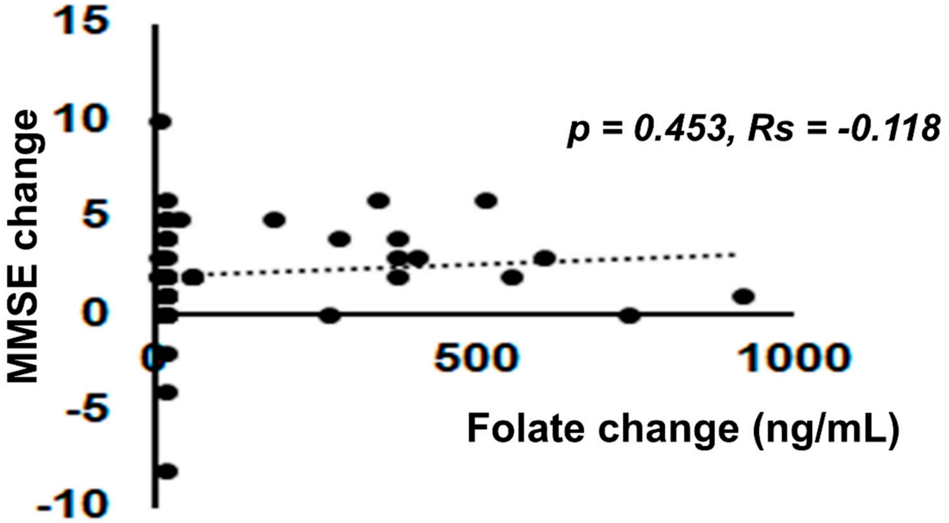

Supplementary Figure S4. MMSE change after folate supplementation and degree of recovery of folate were not correlated. There was no significant correlation between the degree of MMSE change after folate supplementation and degree of recovery of folate (p = 0.453, Rs=-0.118). Spearman's rank correlation coefficient was used because the data deviated from a normal distribution.

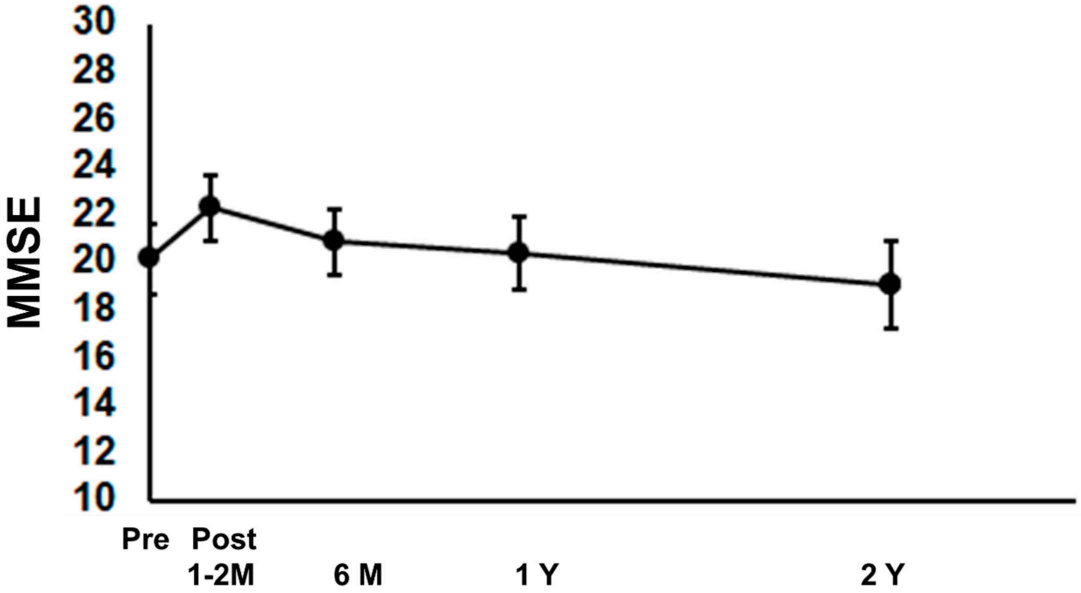

Supplementary Figure S5. Long-term follow-up of Mini-Mental State Examination (MMSE) score.

Long-term follow-up of the MMSE score at 6 months, 1 year and 2 years later by the generalized linear mixed model (GLMM). The decline in the MMSE score was gradual. M=months, Y=Years.

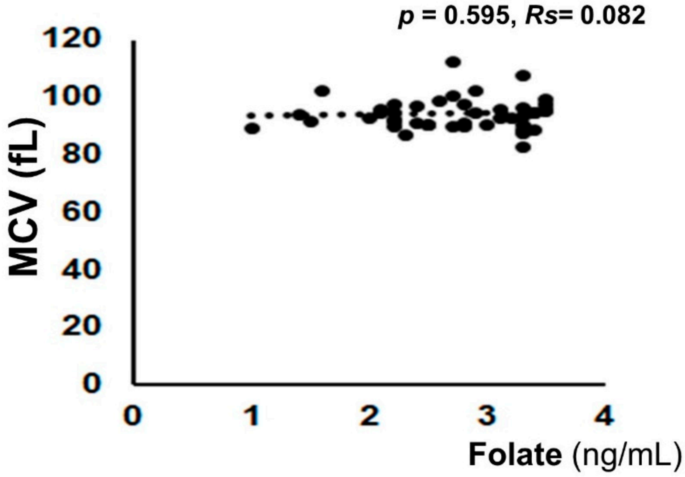

Supplementary Figure S6. There was no correlation between the folate concentration and mean corpuscular volume (MCV). The folate concentration and mean corpuscular volume (MCV) were not significantly correlated ($p = 0.595$, R= 0.082). Spearman's rank correlation coefficient was used for analysis because the folate data deviated from a normal distribution.

Supplementary Table 1 Detailed information of 45 patients.

No.	Age/Sex	Education (Years)	Folate (ng/mL)		VitB12 (pg/mL)	Hcy (nmol/mL)		MCV (fL)	MMSE (years)					Interval (Days)	MRI	
			Pre	Post	Pre	Pre	Post	Pre	Pre	Post	0.5	1	2		VSRAD	Total area
1	74/M	9	1.0	7.6	600	107.2	10.4	89.8	10	20	NA	NA	NA	41	NE	NE
2	77/M	9	1.4	380.0	463	46.6	16.7	94.3	20	24	NA	NA	NA	56	2.04	NE
3	85/F	12	1.5	270.0	715	33.3	14.8	92.1	21	21	NA	NA	NA	56	2.16	NE
4	81/M	12	1.6	8.6	257	74.8	6.2	102.4	17	19	NA	NA	NA	31	NE	NE
5	83/M	9	2.0	382.0	314	22.3	14.4	93.0	18	21	18	NA	NA	49	3.36	6.78
6	84/M	6	2.1	560.0	252	24.8	13.4	96.4	18	20	NA	NA	NA	56	NE	NE
7	86/F	6	2.1	936	868	20.8	5.1	94.7	10	11	NA	NA	NA	47	1.83	15.68
8	86/F	6	2.2	>20.0	870	18.9	8.6	95.0	16	21	21	21	NA	56	0.77	NE
9	77/M	9	2.2	610.0	325	31.4	17.7	98.2	25	28	NA	NA	NA	56	2.50	8.21
10	58/M	12	2.2	>20.0	709	16.9	9.0	90.5	24	25	21	NA	NA	56	NE	NE
11	59/M	12	2.2	61.0	726	27.2	18.2	92.5	20	22	NA	NA	NA	56	5.75	15.68
12	85/M	6	2.2	61.1	2100	21.3	12.1	92.1	18	20	21	21	NA	63	0.30	24.33
13	81/F	6	2.3	380.0	359	19	9.2	87.3	23	25	NA	NA	NA	56	1.72	NE
14	89/M	6	2.4	14.7	517	18.5	11	97.4	28	28	NA	26	27	56	1.89	12.61
15	87/F	9	2.4	18.5	356	33.3	21.6	91.7	15	19	18	17	NA	35	2.47	NE
16	86/F	6	2.5	350.0	326	51.6	19.7	NE	14	20	NA	NA	NA	56	NE	NE
17	82/F	9	2.5	>20.0	369	14.0	7.0	90.8	25	21	20	22	21	28	1.07	5.45
18	78/F	6	2.5	>20.0	>1500	40.2	19.4	90.6	22	28	23	18	17	56	1.60	22.0
19	89/F	6	2.6	410.0	298	19.2	10.2	98.9	14	18	17	15	14	56	1.09	23.02
20	72/F	9	2.7	>20.0	325	9.7	5.4	113.0	29	29	NA	NA	NA	56	NE	NE
21	75/M	12	2.7	>20.0	844	7.5	5.0	90.2	21	21	20	23	18	63	3.00	63.86
22	84/F	6	2.7	>20.0	>1500	5.9	4.6	100.7	23	25	25	24	22	56	1.89	34.95
23	72/M	9	2.8	7.4	273	34.7	16.9	91.7	21	24	24	23	24	28	1.05	1.09
24	81/M	6	2.8	>20.0	361	19.3	12.4	98.0	15	16	17	17	15	35	1.85	18.12
25	72/F	9	2.8	>20.0	>1500	6.9	5.6	90.2	24	25	23	22	20	62	2.09	51.98
26	78/M	6	2.9	>20.0	29.3	20.9	9.5	102.4	24	16	NA	18	NA	42	0.78	18.9
27	68/M	9	2.9	11.0	299	17.3	9.9	95.2	14	16	NA	NA	NA	56	0.70	NE
28	87/M	6	3.0	>20.0	823	29.8	15.9	91.0	19	19	NA	NA	NA	35	NE	NE
29	90/M	12	3.1	17.8	406	39.0	13.6	96.0	23	26	23	NA	NA	56	NE	NE

30	72/M	9	3.1	>20.0	574	12.1	6.6	93.4	16	20	15	16	14	46	NE	NE
31	89/F	6	3.2	520.0	392	17.7	12.6	93.2	14	20	19	13	8	56	0.75	10.96
32	75/M	6	3.3	>20.0	338	13.3	6.7	91.0	19	17	NA	13	11	56	5.37	15.45
33	90/F	6	3.3	40.9	315	11.7	7.2	97.0	13	18	NA	NA	NA	56	5.01	26.8
34	66/M	12	3.3	>20.0	320	23.8	12.1	89.1	18	19	16	18	15	56	3.14	9.2
35	79/M	6	3.3	189.0	354	31.5	11.8	83.2	25	30	29	29	30	56	1.32	15.98
36	72/F	9	3.3	>20.0	378	29.9	11.2	93.7	18	23	21	23	NA	56	2.07	9.35
37	90/F	6	3.3	740.0	393	20.9	10.5	108.0	19	19	19	18	18	56	0.70	23.88
38	87/M	6	3.3	>20.0	1000	24.0	10.4	88.1	22	23	22	19	NA	28	NE	NE
39	84/F	6	3.4	290.0	525	10.6	6.7	88.9	22	26	23	23	26	56	0.75	19.09
40	75/M	12	3.4	920.0	463	19.9	8.1	94.8	26	27	24	28	22	32	NE	NE
41	85/M	9	3.4	20.1	276	13.1	8.6	94.8	26	27	NA	25	27	63	0.52	19.05
42	77/M	9	3.5	>20.0	293	20.1	9.6	98.0	26	28	27	NA	NA	56	0.71	NE
43	85/M	12	3.5	>20.0	402	11.6	8.8	95.3	26	28	28	27	27	28	0.93	0.59
44	83/M	6	3.5	60.6	410	20.6	11.9	99.7	22	24	23	21	NA	56	1.77	11.44
45	72/M	9	3.5	>20.0	410	13.3	6.8	96.0	24	25	24	23	22	56	NE	NE

Data for all 45 patients: age, sex, laboratory tests for folate, vitamin B12, and Hcy, and MMSE score. MCV was performed for 44, and MRI VSRAD was performed for 30.

Abbreviations: Hcy, homocysteine; MMSE, Mini-Mental State Examination; VSRAD, voxel- based specific regional analysis system developed for the study of Alzheimer's disease.

Supplementary Table 2. Comparison of Demographic data

	Included (N=47)	Not included (N=80)	*p*
Age (Mean ± SD)	79.7 ± 7.9	78.8 ± 9.6	0.727
Male sex, n (%)	28 (62.2)	45 (56.3)	0.808
MMSE (Mean ± SD)	20.1 ± 4.7	20.8 ± 5.28	0.485
Folate (Mean ± SD), ng/mL(3.6 - 12.9)	2.7 ± 0.6	2.7 ± 0.6	0.839
Vitamin B12 (Mean ± SD), pg/mL(233 - 914)	558.4 ± 406.5	581.7 ± 475.8	0.677
Hcy (Mean ± SD), (3.7 - 13.5)	25.0 ± 18.0	25.1 ± 23.5	0.986
MCV (Mean ± SD), fL (83.6 – 98.2)	94.6 ± 5.4	94.3 ± 9.8	0.873
VSRAD z-score (Mean ± SD)	1.91 ± 1.37	2.01 ± 1.41	0.868

Comparison of demographic data between patients included in this study (N=45) and those not included (N=80).

Abbreviations: SD, standard deviation; MMSE, Mini-Mental State Examination; Hcy, homocysteine; (), normal range; VSRAD, voxel-based specific regional analysis system developed for the study of Alzheimer's disease
